# Natural Killer T Cell Diversity and Immunotherapy

**DOI:** 10.3390/cancers15245737

**Published:** 2023-12-07

**Authors:** Eduardo I. Tognarelli, Cristián Gutiérrez-Vera, Pablo A. Palacios, Ignacio A. Pasten-Ferrada, Fernanda Aguirre-Muñoz, Daniel A. Cornejo, Pablo A. González, Leandro J. Carreño

**Affiliations:** 1 Millennium Institute on Immunology and Immunotherapy, Santiago 8330025, Chile; eitognar@uc.cl (E.I.T.); crgutierrez@uchile.cl (C.G.-V.); pablopalacios@uchile.cl (P.A.P.); ipastenf@uc.cl (I.A.P.-F.); amunoz.fer27@gmail.com (F.A.-M.); daniel.cornejo@uc.cl (D.A.C.); 2 Facultad de Ciencias Biológicas, Pontificia Universidad Católica de Chile, Santiago 8331150, Chile; 3 Programa de Inmunología, Instituto de Ciencias Biomédicas, Facultad de Medicina, Universidad de Chile, Santiago 8380453, Chile

**Keywords:** natural killer T cells (NKTs), infectious disease, cancer, immunotherapy, antigen presentation, inflammation

## Abstract

**Simple Summary:**

Cell-based oncotherapies are gaining considerable terrain in the last years thanks to the development of safe and promising treatments against cancer. A better understanding of the roles and functional capacities of cellular immune components displaying antitumor activity will likely potentiate these encouraging results. In this review, we summarize the properties and uses of a particular type of immune cells that recognize lipid-derived determinants presented on the cell surface. Such immune cells, known as invariant Natural Killer T (iNKT) cells have the potential to significantly favor anti-tumoral adaptive immune responses. Currently, they are being extensively evaluated in preclinical settings and may soon reach clinical trials to ameliorate anti-cancer treatments.

**Abstract:**

Invariant natural killer T cells (iNKTs), a type of unconventional T cells, share features with NK cells and have an invariant T cell receptor (TCR), which recognizes lipid antigens loaded on CD1d molecules, a major histocompatibility complex class I (MHC-I)-like protein. This interaction produces the secretion of a wide array of cytokines by these cells, including interferon gamma (IFN-γ) and interleukin 4 (IL-4), allowing iNKTs to link innate with adaptive responses. Interestingly, molecules that bind CD1d have been identified that enable the modulation of these cells, highlighting their potential pro-inflammatory and immunosuppressive capacities, as required in different clinical settings. In this review, we summarize key features of iNKTs and current understandings of modulatory α-galactosylceramide (α-GalCer) variants, a model iNKT cell activator that can shift the outcome of adaptive immune responses. Furthermore, we discuss advances in the development of strategies that modulate these cells to target pathologies that are considerable healthcare burdens. Finally, we recapitulate findings supporting a role for iNKTs in infectious diseases and tumor immunotherapy.

## 1. Introduction to Unconventional T Cell Subtypes, iNKTs, and CD1d Interactions

The first responders in an inflammatory process upon pathogen infection are usually innate immune cells, which are fundamental for initiating, propagating, and regulating adaptive immune responses [1,2,3]. Their role in the adaptive response may go on to determine the outcome of infection resolution and the establishment of immunological memory against pathogens [1,4]. This is accomplished by their influence in modulating the activation and differentiation of T cells and B cells due to their rapid infiltration into the site of infection and response, accompanied by tweaking effector molecules (i.e., surface markers and cytokine secretion) over immune cells, which exerts immunomodulatory effects [1,4]. However, these innate cells may also be involved in exacerbated inflammatory responses in different immunological contexts, such as pathogen infections, self-reactive autoimmune disorders, and cancer, leading to poor pathogen clearance and detrimental outcomes for the host [5,6].

Among the varied existing innate immune cell populations, there are monocytes, macrophages, granulocytes, and polymorphonuclear (PMN) cells consisting of neutrophils, eosinophils, mast cells, and basophils; there are also dendritic cells and innate lymphoid cells (ILCs) [7,8,9,10,11,12]. ILCs have the particularity of being lymphocytes that have activating and inhibiting receptors and are susceptible to cytokine stimulation. However, they are functionally independent of antigen recognition due to the lack of adaptive antigen receptors [13,14]. These cells have distinct cytokine profiles and differential expression of key transcription factors (TFs), and for ILCs, they are classified according to their capacity to respond to microorganisms, much like T helper cells perform in the context of adaptive immune responses [14,15,16].

ILCs are subdivided into three subgroups: ILC1, ILC2, and ILC3. Interferon gamma (IFN-γ)- and tumor necrosis factor alpha (TNF-α)-producing group 1 innate lymphoid cells (ILC1s) are dependent on the expression of T-box transcription factor 21 (TBX21, T-bet) [17,18]. ILC1s also include cytotoxic ILCs, also known as natural killer (NK) cells, which express Eomesodermin (Eomes) and altogether react to intracellular pathogens such as bacteria and viruses [19,20]. IL-4-, IL-5-, and IL-13-producing group 2 innate lymphoid cells (ILC2s) are dependent on the transcription factors GATA binding protein 3 (GATA3) and RAR-related orphan receptor alpha (RORα) and respond to extracellular parasites and allergens [21,22]. IL-17- and IL-22-producing group 3 innate lymphoid cells (ILC3s) are dependent on the expression of the transcription factor RAR-related orphan receptor γt (ROR-γt) and mainly respond to the presence of extracellular pathogenic bacteria and fungi [23,24].

**Figure 1:**
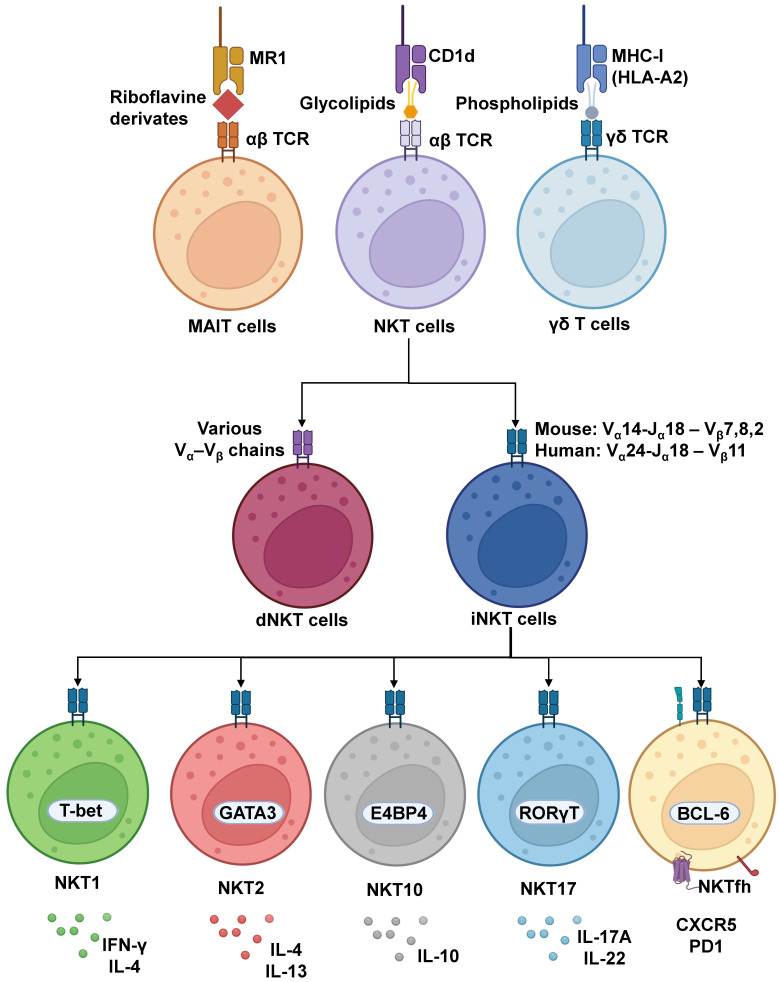
**Innate-like T cell and NKT subsets**. Innate-like T cells are classified as riboflavin-recognizing mucosal-associated invariant T (MAIT) cells, glycolipid-activated NKT cells, and phospholipid-responding gamma-delta T cells that overall differ in the residing tissues of preference and antigen specificity. Nonetheless, these cells show restrictions on their antigens and the nature of antigen recognition. They also display severely reduced plasticity. However, dNKT cells are somewhat of an exception due to their variable TCR alpha chains, which lead to increased antigenic flexibility. Nevertheless, their modulation is poorly understood. Invariant NKT cells have been described to behave similarly to CD4^+^ T helper cells due to the various subset phenotypes they can acquire after activation, producing cytokine secretion patterns characteristic of particular subsets: NKT1 (IFN-γ), NKT2 (IL-4), NKT17 (IL-17), as well as regulatory NKT10 (IL-10) and T follicular helper NKTfh cells. Figure created with BioRender.com.

On the other hand, there are innate-like T cells, which are more closely related to the adaptive immune response [25] (Figure 1). These cells mostly develop in the thymus, although some may develop extrathymically [26] from progenitors expressing αβ and γδ T cell receptors (TCRs), similar to conventional T cells that express αβ TCRs and recognize antigens presented on major histocompatibility complex (MHC) molecules. However, innate-like T cells differentiate themselves from T cells by having characteristics of innate immune cells, although they may initiate and connect with the adaptive host immune response through their effector and regulatory functions [27,28,29]. These peripheral innate-like T cells that are also considered unconventional T cells, with the exception of diverse natural killer T cells (dNKTs), have limited TCR diversity compared to conventional T cells [30,31,32], and unlike such cells, they recognize antigens that are non-peptide in nature, including vitamin B (riboflavin) metabolites, phosphoantigens, and lipid-based antigens [33]. Additionally, a common trait of these unconventional T cell subpopulations is that they rapidly secrete cytokines, and some subsets co-express surface antigens related to natural killer (NK) cells, like CD161 for mucosal-associated invariant T (MAIT) cells or NKG2D for NKT and γδ T cells [32,34,35], strongly influencing an immune response after their activation. These cells are known as CD1-restricted T cells: natural killer T (NKT) cells, MHC-related 1 (MR1)-restricted T cells: MAIT cells, and γδ T cells [36].

MAIT cells are a subset displaying a semi-invariant TCR that is activated in mammals by riboflavin derivatives loaded onto the highly conserved major histocompatibility complex class I-related (MR1) molecule [37,38]. Their role in infectious diseases, along with their antibacterial and antifungal effects, has been reported [39,40]. Once active, MAIT cells produce cytotoxic substances in order to counteract pathogens, similar to NK cells [41,42,43], and they participate in tissue repair to procure homeostasis [44,45,46] and mount strong cytokine responses, consequently affecting the involvement and functions of other immune cells [47,48]. Nonetheless, unchecked activation of MAIT cells, which may also be achieved by IL-2, IL-12, and IL-18 stimulation, can potentiate diverse adverse effects, such as cytokine storms [49], tissue fibrosis [50], and tumor cell metastasis, among others [51,52]. On the contrary, due to infections and aging, reduced accounts of this subset may have detrimental health consequences as well, such as dysregulated tumor immunity, increased risk of developing intestinal bowel disease (IBD), or poor resolution of hepatic infections [32,53,54].

On the other hand, γδ T cells are a unique cell subset that expresses a γδ TCR, located predominantly in epithelial mucosal tissues and infiltrate tumor tissues. They are capable of recognizing tumor-associated antigens and attacking tumor cells via cell-to-cell interactions [55,56,57]. These cells have been described as acting without major histocompatibility complex (MHC) restrictions; as such, some antigens may be identified in the MHC-I context but with no peptide recognition [58]. In addition, the most abundant γδ TCR population recognizes phosphoantigens of bacterial nature [59], while some γδ T cells with CD1d-restricted TCRs may recognize lipids [60]. Incidentally, although initially considered key against cancer, some populations of γδ T cells may secrete IL-17, suppressing anti-tumoral effects and promoting tumor immune evasion [61,62,63].

Lastly, NKT cells, as their name implies, share features of NK cells, as they possess several NK cell receptors [64] and T cells [65], due to having a TCR that recognizes lipid-based antigens. They are known for their innate-like response to stimuli and are hailed for their specific expansion, similar to that observed in an adaptive cellular response [66,67,68]. These cells are also subdivided into two major subclasses: type 1 NKT cells (iNKTs), which are more frequent in mice than humans, possess a unique αβ TCR composed of an invariant α-chain (Vα14–Jα18 in mice, Vα24–Jα18 in humans) that is associated with limited variants of β-chains (Vβ8, Vβ7, and Vβ2 in mice, Vβ11 in humans) [69,70]; whereas type 2 NKT cells have a more diverse repertoire of TCRs and are known as diverse NKT cells (dNKTs), due to other available Vα and Vβ chains, and are more frequent in humans than in mice [71,72]. In particular, like T cells, iNKTs arise from immature CD4^+^CD8^+^ precursor T cells in the thymus during T cell differentiation and ultimately express common surface markers, such as CD69, CD44, CD62, CD11a, and CD122 [73]. To achieve recognition of lipid antigens presented to T cells, APCs employ CD1 antigen-presenting molecules classified in group 1 (CD1a, CD1b, and CD1c), group 2 (CD1d), and the soluble lipid-editing CD1e [74,75]. Importantly, although CD1a through e are found in humans, mice are restricted to CD1d [76]. On the other hand, studies have also found similarities between the human and guinea-pig CD1 systems, since guinea-pigs have an extended group 1 CD1 gene family, with human homologues of CD1b and CD1c proteins being expressed at similar levels on APCs such as monocyte-derived DCs [77]. The same is true between human and rabbit CD1 systems, as rabbits possess multiple CD1 loci, with a particular one being closely related to the human CD1b gene [78]. These molecules have different binding cleft structures to display antigens of distinct types and extensions and have heterologous distribution: only Langerhans cells express high amounts of CD1a, myeloid cells have high expression of CD1b, and B cells express CD1c [75,79]. CD1d is unique since it can be found broadly distributed on the surface of hematopoietic and nonhematopoietic cells, including epithelial cells, monocytes, and, importantly, macrophages, B cells, and DCs. CD1d can recognize shorter antigens than CD1b and even slight alkyl chain variations in the lipidic antigens loaded, which may affect antigenic potency [80], to induce activation of NKTs and γδ T cells [60,81]. Therefore, both iNKTs and dNKTs have TCRs that are restricted to interactions with the MHC-I-like molecule CD1d [71,82]. As such, numerous molecules able to bind to CD1d have been assessed for their effects on iNKT activation [68,83,84], which will be discussed below.

Altogether, the various existent innate cell subtypes act in response to a wide range of conditions and stimuli. Importantly, the exposure to different signals in the inflammatory milieu exerts pressure over the plasticity of these cells, causing them to adapt their transcriptional and proteomic profiles, which together will modulate downstream immune responses [17]. In this review article, we will describe recent findings and strategies focusing on the characteristics of NKT cells, which are being explored in cancer immunity and infectious diseases.

## 2. Role of iNKTs in Innate and Adaptive Immune Responses

NKT cells develop in the thymus, undergoing positive selection by thymocytes, antigen-specialized phenotypes, and functional maturity, allowing them to later on respond quickly to TCR and cytokine signaling, besides their capacity to elicit immediate effects through the production of cytokines [85]. Once iNKT cells develop in the thymus, these cells migrate to the periphery and are distributed heterogeneously among both immune and non-immune organs, including the thymus, spleen, lymph nodes, small intestine, lung, liver, and fat [68]. Although NKT cells can be located in most tissues, they are usually most abundant in the liver, spanning between 20% and 50% of the available T cell populations [86,87]. From there, NKT cell distribution decreases, being found in the thymus at about 10–20%, bone marrow at 20–30%, and in the lymph nodes and peripheral blood at approximately 0.1% to 0.5% of total T cells [88].

As NKT cells are innate type cells with morphologies and lineage functions that are related to NK cells and T cells, particularly through the recognition of glycolipid antigens presented by CD1d in cells such as DCs and B cells [65], NKT cells can fulfill various host defensive roles [89], namely participating in establishing and regulating innate and adapting immunity. The recognition of lipid-derived antigens by NKTs has made it possible to determine the consequences of activation of these NKT cells in various contexts. In a primary step, antigens are captured by membrane receptors of antigen-presenting cells, requiring support from microsomal triglyceride and saponin transfer proteins, which in turn participate in the re-internalization of the CD1d-antigen receptor and its mobilization into the vesicular trafficking system [64]. Once inside the vesicles, antigens are processed with the help of sphingolipid-activating proteins. Afterwards, processed antigens are loaded onto CD1d thanks to molecules such as CD1e, which are responsible for coupling the lipids to CD1d molecules, which will be presented on the membrane surface [64]. Once these cells are activated by macrophages, dendritic cells, B cells, or neutrophils that secrete pro-inflammatory cytokines such as IFN-γ, IL-12, and IL-18 [73], iNKT cells, through the TCR receptor, will recognize the CD1d-antigen complex or allow for the interaction through the CD40-CD40L complex [68]. Afterwards, iNKT cells modulate the immune response through their particular capacity to secrete a wide range of cytokines following activation [90], such as interferon (IFN)-γ, tumor necrosis factor (TNF)-α, interleukin (IL)-2, IL-4, IL-5, IL-13, IL-10, IL-17A, IL-22, and granulocyte macrophage colony-stimulating factor (GM-CSF) [91,92,93]. In turn, the cytokines released by NKT cells enable the activation of B cells, initiating a signaling cascade that promotes the differentiation of memory B cells, or antibody-secreting plasma cells [94]. Furthermore, studies support that NKT cells could activate B cells, where these activated NKT cells would acquire a follicular helper program (NKTfh), characterized by CXCR5, CD4, and PD1 expression, through induction of the transcription factor BCL-6 and CD28 co-stimulation [95]. Thereafter, these cells would enter the B cell follicles in a manner dependent on the chemokine receptor CXCR5 to form long-lasting conjugates with B cells, promoting the formation of germinal centers and antibody production [96].

Noteworthy, CD1d-derived antigen presentation induces the activation of iNKTs, where depending on the response, either an effector or suppressor immune response, pro-inflammatory or anti-inflammatory cytokine production is generated, respectively [97]. The similarities of iNKTs with T cells have allowed for a convention in referencing their polarization profiles. Indeed, the secretion of cytokines resulting from the activation of NKT cells has led to the determination of the subtypes these cells may acquire, in agreement with their cytokine secretion pattern, different surface expression markers, and main transcription factors [68].

Overall, the majority of NKT cells differentially express promyelocytic leukemia factor (PLZF) to trigger differentiation and maintain expression in their mature effector subsets [98], and as such, iNKT cells are subdivided into subsets, the traditional ones being NKT1, NKT2, and NKT17, based on their similarity to the cytokine profiles produced by Th1, Th2, and Th17 lymphocytes, respectively, in addition to a more recently described IL-10-secreting iNKT10 subset [68,99]. In particular, NKT1 cells have been related to a cytotoxic function due to expressing the transcription factor T-bet and secreting IFN-y and IL-4 after activation. On the other hand, NKT2 cells secrete IL-4 and IL-13, which have been proposed to maintain homeostasis by reducing inflammatory conditions and promoting tissue repair [68,100]. Meanwhile, IL-17 and IL-23-producing NKT17 cells have been implicated in both host defense against fungal infections and in the pathogenesis of various autoimmune diseases, such as asthma and psoriasis, as well as graft-versus-host disease [101,102,103]. Lastly, NKT cells may acquire a NKT10 phenotype, which is akin to IL-10-producing regulatory CD4^+^ T cells [93]. These NKT10 cells do not express the PLZF transcription factor but rather express E4 promoter-binding protein 4 (E4BP4) [104] and FOXP3 in the presence of TGF-β [105]. These iNKT subsets have been found to be more prevalent and to participate in adipose tissue homeostasis [106], but they also function in controlling intestinal inflammatory CD4 T cells [107]. However, overall iNKT commitment toward these profiles is somewhat plastic, as they are nevertheless able to produce other cytokines upon their polarization (such as IL-4 for NKT1 cells) [108]. Upon stimulation, a characteristic early secretion of IL-12 and a later secretion of IFN-γ and IL-4 have also been reported [81].

Although research on NKT cell natural function considers their ability to mount adaptive immunity through the recognition of glycolipid antigens from pathogenic bacterial agents, such as *Streptococcus pneumoniae* and *Clostridium difficile*, among others [96], a prototypical ligand of CD1d that has been thoroughly studied has been α-galactosylceramide (α-GalCer or KRN7000), which is a synthetic glycolipid derived from a glycosphingolipid from the marine sponge *Agelas mauritianus*. This compound was originally found to polarize NKTs towards NKT1 and NKT2 [109], although it was later found to stimulate NKTs to acquire the other phenotypes, NKT17 and NKT10, suggesting the broad potential of α-GalCer in activating NKT cells [93,103]. Several analogues of this molecule have been assessed since its discovery to determine their effects on different diseases, such as cancer [82,110,111]. Some of these synthesized analogues are α-C-GalCer in nature and produce Th1-type polarized responses. However, by introducing certain modifications in the structure of this molecule, particularly changes in the sugar and lipid chains [109], other α-GalCer analogues have been synthesized, such as OCH, which tends to trigger Th2 responses [64]. Moreover, the molecules named 7DW8-5, AH03-1, and AH10-7 are capable of inducing potent NKT1 responses, even stronger than those induced by α-GalCer in in vivo mouse models, producing significant IFN-γ secretion [84,112,113]. This type of response could be expected to elicit increased antiviral immunity against widespread pathogens [84]. It may also elicit significant immunity against cancer [114].

Overall, it has been established that α-GalCer and its analogues could induce the activation of NKT cells, causing the formation of direct B cell-NKT cell conjugates to help B cells in the generation of antibodies, whereas their indirect participation resulting from their interaction with dendritic cells could potentiate their immunomodulatory effects [96].

## 3. α-Galactosylceramide and Its Derivatives as Modulators of iNKT-Dependent T Helper-like Responses

**Figure 2:**
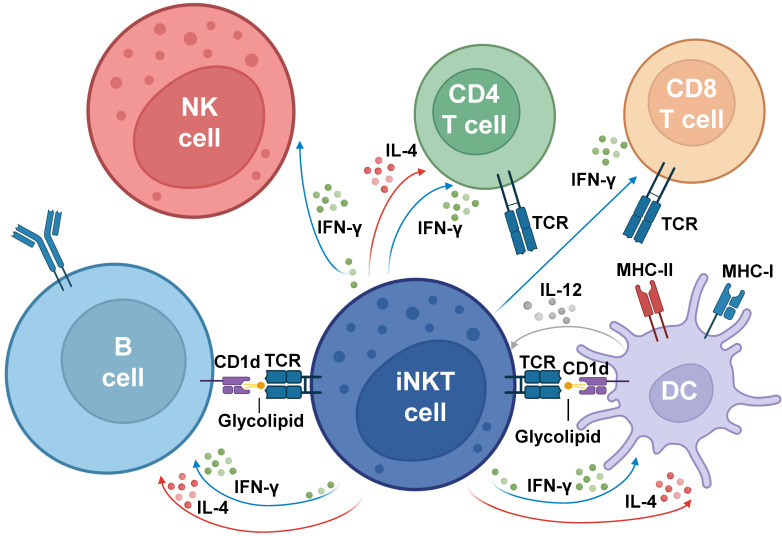
**Model for NKT cell transactivation of other immune cells**. The contribution of NKT cells to the activation of T cells, B cells, and antigen-presenting cells (APCs) is illustrated in this figure. The first step requires that NKT cells recognize, through their TCRs, glycolipids loaded on CD1d molecules on the surface of DCs or B cells. In response to antigen recognition, NKT cells mediate a rapid secretion of cytokines with the release of high quantities of IL-4 and IFN-γ, among other cytokines. Once activated, NKT cells may differentiate into particular phenotypes, as described in Figure 1, and indirectly modulate the function of NK cells, CD4^+^, and CD8^+^ T cells, whereas they are also able to stimulate DCs and promote the differentiation of B cells. Figure created with BioRender.com.

After the activation of iNKT cells, the cytokines secreted by these cells induce the recruitment and transactivation of multiple immune subsets, including neutrophils [115], natural killer cells [116], macrophages [117], dendritic cells [118], as well as CD4^+^ and CD8^+^ T cells [119], and B cells [120], thus revealing iNKT cells as a functional bridge between innate and adaptive immune responses (Figure 2).

Multiple studies have highlighted a significant role for glycolipid-stimulated iNKT cells as potent tools for immunotherapeutic strategies against infectious diseases and cancer. As such, the use of α-GalCer and other compounds with similar chemical structures has been considered as vaccine adjuvants with significant effects against different pathologies [121]. Early studies demonstrated that the activation of iNKT cells mediated by the in vivo administration of α-GalCer led to increased production of IFN-γ and upregulation of the activation marker CD69 on CD4^+^ T cells [122]. A similar methodology was used by Singh and collaborators, demonstrating that concomitant administration of α-GalCer and chicken ovalbumin (OVA) protein induced a significant antigen-specific CD4^+^ T cell response in animals [123]. Additionally, it has been demonstrated that strong antigen-specific CD4^+^ T cell responses are dependent on the maturation of dendritic cells induced by α-GalCer, increasing the priming of CD4^+^ T cells [83]. Further investigations have demonstrated the adjuvant capacity of α-GalCer in the context of different infectious diseases, including viruses, bacteria, and parasites, through vaccines, including some based on subunits, semisynthetic, attenuated DNA, and recombinant viral vectors admixed with α-GalCer, among others [124].

Regarding parasitic infections, the administration of α-GalCer with different *Plasmodium* spp.-derived antigens has been demonstrated to elicit both IFN-γ production and malaria-specific CD4^+^ T cells, leading to an increased anti-malaria immunity in mice [125]. On the other hand, mice immunized with DNA encoding the *Leishmania infantum* p36 protein along with α-GalCer and a further boost with a vaccinia virus-encapsulated p36 encoding plasmid promoted the relative expansion of spleen-derived antigen-reactive CD4^+^ T cells, as well as granzyme B-producing CD4^+^ T cells, increasing the protection against *L. major* infection, as evidenced by an increase in size lesion development and parasite burden when CD4^+^ T cells were depleted [126].

Despite the therapeutic potential of α-GalCer in different infectious diseases, as mentioned above, iNKT cell activation mediated by this compound leads to the concomitant secretion of multiple cytokines, some of which have opposing functions, limiting the effectiveness of α-GalCer as an immunomodulator and potentially causing the development of other diseases [127]. Furthermore, the upregulation of a particular pathway could cause the downregulation of other pathways, leading to reciprocal inhibition [128]. Furthermore, it has been demonstrated that a single administration of α-GalCer can lead to long-term anergy in iNKT cells [129], as well as inducing liver injury in mice and liver toxicity in humans [130]. This evidence has led to the generation of α-GalCer-derived compounds that stimulate Th1- or Th2-biased responses derived from the activation of iNKT cells [131].

The α-GalCer derivative 7DW8-5 is characterized by having a shorter acyl chain that terminates in a fluorinated benzene ring [132] and induces a Th1-like response by iNKT cells, promoting an increased response against viral antigens expressed by recombinant *Mycobacterium bovis* Bacillus Calmette-Guérin (rBCG). This was evidenced through the enhanced priming of Gag-specific CD8^+^ T cells in mice that received the simian immunodeficiency virus antigen Gag expressed in a rBCG that had incorporated the glycolipid 7DW8-5 [133]. Furthermore, administration of an HIV vaccine approach along with 7DW8-5 caused an increased p24-specific CD8^+^ T cell response [132]. Moreover, it was observed that mice immunized with a recombinant adenovirus expressing a *Plasmodium yoelii* CS protein admixed with 7DW8-5 promoted an increased malaria-specific CD8^+^ T cell response as compared to α-GalCer adjuvant stimulation [108]. In a similar context, mice immunized with a *Plasmodium yoelii* circumsporozoite protein along with 7DW8-5 increased the percentage of central memory CD4^+^ T cells and effector memory CD8^+^ T cells, promoting malaria-specific immunity [112]. On the other hand, by using *Salmonella* Pathogenicity Island 2 (SPI2) and its type III secretion system (T3SS) to deliver tumor-associated antigens along with 7DW8-5-induced IL-12 and IFN-γ secretion to promote anti-tumor Th1 response and cytotoxic T cells [134]. Finally, administration of 7DW8-5 alone to mice promoted an anti-viral state that prevented SARS-CoV-2, respiratory syncytial virus, and influenza virus infection, each by mechanisms yet to be defined [135].

OCH, a sphingosine-truncated α-GalCer analog, which promotes a prominent Th2-like cytokine secretion upon the activation of iNKT cells [136], has been shown to have beneficial effects against *Trichuris muris* infection in mice when administered along with its recombinant WAP49 protein, leading to an absolute increase in IL-4-producing CD4^+^ T cells, promoting a robust and functional Th2 response [137].

Notwithstanding the fact that, up to date, the generation of novel α-GalCer derivates is an active field of study, most of the current research is mainly focused on the evaluation of the biological activity of such compounds in vitro and in vivo, yet functional studies in the context of different pathologies regarding their effectiveness and related mechanisms of action remain to be determined.

## 4. Role and iNKT Modulation Strategies in Infectious Diseases and Hepatocarcinoma

As mentioned earlier, iNKT cells are particularly important for bridging innate and adaptive immune responses, which derives from the potential of broad-spectrum protection against pathogens to maintain health. This is especially significant because these cells reside in different tissues, allowing them to activate and respond rapidly when encountering antigens. However, as the specific role of these subsets in various disease contexts is not fully understood, the knowledge acquired while studying their implications in infectious diseases has been a focus of research that has enabled the development of strategies to harness their vast immunomodulatory potential, not only to mitigate the harm caused by pathogens but also in immunotherapy approaches.

As such, it has been observed that in bacterial infections, the study of murine models of TB has enabled the identification of microbial CD1d-restricted glycolipid antigens, which promote iNKT cell activation and induce transactivation of other immune cells, although IL-12 and IL-18-mediated activation also occur [138]. The resulting cellular targets of iNKT cell-mediated transactivation include DCs through GM-CSF production and IFN-γ, which in turn results in increased cytotoxic activity by CD8^+^ T cells, a process potentiated by IFN-γ [139]. It has been observed that intracellular cytokine analysis of T CD4^+^ responses to sublingual immunization with either Mtb-derived proteins Ag85B and ESAT-6, along with α-GalCer, caused an increase in the percentage of IFN-γ and IL-2-producing CD4^+^ T cells in organs such as the spleen, lungs, and cervical lymph nodes, observing similar results when mice were further challenged with Mtb after subunit vaccination [140]. Indeed, incorporating α-GalCer into rBCG vectors has allowed for increased CD8+ T cell response with concomitant enhanced protection from *Mycobacterium tuberculosis* (Mtb) infection [141]. On the other hand, a liposomal vaccine formulation against *S. pneumoniae* infections, consisting of polysaccharide PS-II derived from its serotype 14, along with PBS57, promoted higher antibody titers, together with IgG class-switched antibodies and memory responses [96], highlighting the role of iNKT cells in the induction of cellular processes typically absent in the response against T-independent antigens. Furthermore, *Haemophilus influenzae*-specific protective immunity through intranasal immunization with outer membrane proteins P6 and α-GalCer led to a potent P6-specific CD4^+^ T cell response, mediated by the production of different cytokines such as IFN-γ, IL-2, IL-4, IL-6, IL-10, TGF-β, and IL-17 [142,143].

Regarding virus infections, it has been shown that by using transgenic mouse models and adenoviral HBV expression, iNKT cell activation is achieved through α-GalCer administration, which is capable of promoting systemic IFN-γ production together with IFN-α/β that inhibits viral replication [144]. Moreover, a combined strategy of α-GalCer with the naIAV vaccine evaluated in a murine model was able to promote enhanced antigen-specific CD8^+^ T cell memory response, which correlated with protection from a subsequent challenge with a heterologous influenza virus strain [145,146].

Immunization with a dual-promoter DNA vaccine pADVAX-e/g, encoding human immunodeficiency virus 1 (HIV-1) *env* and *gag*, admixed with α-GalCer, caused an increased number of Env- and Gag-specific IFN-γ-producing CD4^+^ T cells, while the use of α-GalCer on a further DNA prime-DNA boost regimen led to an increased number of Gag-specific IFN-γ-producing CD4^+^ T cells, providing evidence that α-GalCer could promote a robust cellular immune response against HIV infection [147]. Furthermore, the sublingual administration of the C-clade envelope HIV protein gp140 concomitantly with α-GalCer produced a considerable increase in gp140-specific IFN-γ-producing CD4^+^ T cells, and the addition of TLR-9-stimulating adjuvant CpG-ODN led to a further increase in the aforementioned cells [148]. Furthermore, sublingual immunization with an adenovirus vector encoding the HIV envelope antigen (HD-Ad HIV-env) along with α-GalCer resulted in increased percentages of IFN-γ-producing CD4^+^ T cells, as well as elevated percentages of antigen-specific IL-2^+^, IFN-γ^+^, and IL-2^+^/IFN-γ^+^ effector and central memory CD4^+^ T cells [149]. Lastly, glycolipid agonists have been used in the context of SARS-CoV-2 infection, demonstrating that iNKT cell activation using α-GalCer overcomes immune suppression, improving mouse survival [150]. Indeed, prophylactic administration of 7DW8-5 prevented SARS-CoV-2 infection, including delta and omicron variants, and among cytokines promoted by glycolipid administration, IFN-γ was highly upregulated, which correlated with NKT1 expansion in lung MNCs after nasal administration, suggesting its possible role during viral infection [135]. Moreover, the conjugation of the receptor-binding domain (RBD) of the viral spike protein derived from the SARS-CoV-2 virus, along with α-GalCer, promoted an increase in SARS-CoV-2-specific IFN-γ^+^, TNF-α^+^, and IFN-γ^+^/TNF-α^+^ CD4^+^ T cells in comparison to the groups that were immunized with a mix of the RBD protein and α-GalCer [151], generating a novel candidate for a COVID-19 vaccine based on the immunomodulatory properties of iNKT cells. Detailed information regarding iNKT cells in other infectious diseases, including human respiratory syncytial virus (hRSV) and herpes simplex virus (HSV), has been addressed in other reports [152,153,154,155,156,157,158,159].

Comparing immune and non-immune organs, iNKT cells are predominantly located in the liver, especially since in mice, as stated earlier, they occupy the bulk of the intrahepatic T cell populations, whereas for humans, about 3-5% of T cells are NKT cells [68,160,161,162]. The liver is part of the digestive system and plays an essential role in the metabolism and elimination of toxic substances; therefore, a failure in this organ is a life-threatening condition. Hepatocytes express CD1d and are responsible for controlling the number of hepatic NKT cells, which is crucial for maintaining homeostasis in inflammation control and pathological contexts like schistosomiasis [163,164]. The murine liver is highly enriched in the NKT1 subset [165], and interestingly, it has been reported that mice lacking the CXCR6 chemokine receptor, responsible for iNKT cell localization in the liver and spleen, have a lower frequency of liver iNKT cells and reduced production of IFN-γ, which make them more susceptible to infection [166,167,168].

**Figure 3:**
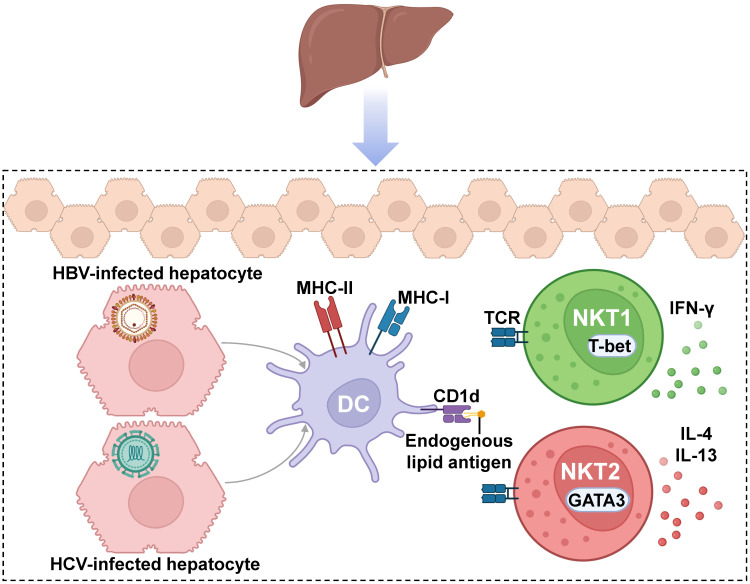
**Invariant Natural Killer T cells (iNKTs) in liver infectious diseases.** In hepatitis virus infections, infected hepatocytes are phagocyted by antigen-presenting cells (APCs), such as dendritic cells, which present lipid antigens loaded on CD1d molecules to activate NKT cells in a TCR-dependent manner. In this context, iNKT cells typically mount an NKT1 response after activation. However, during HBV infection, iNKTs may also respond by secreting IL-4 and IL-13 cytokines, which have been linked to the type 2 CD4^+^ T helper cell-like phenotype NKT2. Figure created with BioRender.com.

The most common liver infections are those carried out by viruses, such as hepatitis A virus (HAV), HBV, HCV, HDV, and HEV [169]. Both HBV and HCV chronic infections are one of the most common causes of liver cirrhosis and liver cancer [170,171]. HBV hepatocyte infection leads to endogenous lipid antigen presentation, which activates NKT cells (Figure 3). When either NKT cells or endogenous lipid presentation are abrogated (Jα18-KO, CD1d-KO, or H-Mttp-KO), activation of virus-specific T and B cells is reduced, affecting anti-viral protection [172]. On the other hand, in a transgenic model of acute hepatitis B virus infection, it was observed that the early response of a nonclassical NKT cell subset, which is CD1d-restricted but does not react to α-GalCer, causes liver injury, likely contributing to viral pathogenicity [173]. Despite the clear role of NKT1 cells and IFN-γ in HBV viral infection, it has been reported that HBV also promotes IL-4 and IL-13 production by iNKT cells, leading to the activation of hepatic stellate cells (HSCs), which are associated with the progression of liver fibrosis. Although the NKT subset has not been specifically defined, this profile could suggest the participation of the NKT2 cell subset [174,175]. On the other hand, liver NK1.1^−^ iNKT cells have been reported to produce IL-17, although their role is associated mainly with airway immunity, which will be addressed later [176]. Moreover, a study determined that iNKT cells in individuals with chronic hepatitis B were substantially reduced, coinciding with increased Fas and FasL expression, but showed increased CD1d and were hyperreactive to α-GalCer stimulation, as observed by the upregulation of CD69, CD38, and HLA-DR [177]. However, these cells were deficient in CD1d-dependent production of IFN-γ, which could only be recovered after exogenous addition of IL-2 and IL-15, implying dysregulated activation of iNKT cells could be associated with disease progression during chronic HBV infection [177].

Regarding HCV, clinical studies of patients with chronic infection have reported controversial data, since on the one hand it has been reported that αβ CD4^+^ T cells were expanded, while CD56^+^ αβ T cells and Vα24^+^ T cells were depleted during chronic infection as compared to control patients, and for CD4^+^ T cells they were mainly IFN-γ producers [178]. A similar study analyzing NKT cell populations in patients with chronic hepatitis C infections without receiving antiviral treatment showed no changes in iNKT cells (CD3^+^ CD56^+^ Vα24^+^ Vβ11^+^). However, there was an increased number of dNKT cells (CD3^+^ CD56^+^ Vα24^−^ Vβ11^−^) when compared to control individuals, and leukocytes from infected patients showed reduced expression of CD1a, CD1c, and CD1d, although it was not clear whether this contributed to the chronic state of the infection [179]. Additionally, it has been reported that adoptively transferred human PBMC-derived iNKT cells, when treated with IFN-α, potentiate IFN-γ production by NKT cells, inhibiting viral replication. However, when PBMCs were depleted of iNKT cells prior to cell transfer, this effect was reversed [180].

Importantly, persistent infections with particular types of viruses, bacteria, and parasites have been identified as potential factors that increase the likelihood of cancer development. Indeed, these infections have been reported to contribute to the cancer process through various mechanisms, including inducing chronic inflammation, promoting genetic mutations, or disrupting regulatory processes governing cell growth and division [181,182]. Indeed, approximately 25% of the factors contributing to the development of cancer are attributed to infectious diseases and chronic inflammation [183].

Considering that iNKT-based therapies against hepatic viruses could help prevent the development of associated illnesses, such as hepatic fibrosis or cirrhosis, which could ultimately result in common types of liver cancer such as hepatocellular carcinoma (HCC), the involvement of NKT in such tissues should be studied [184,185]. Importantly, the tumor microenvironment (TME) constitutes a significant barrier to implementing immunotherapy strategies, hindering the proper functioning of different components of the immune system, such as iNKT cells. Indeed, the TME can act as an obstacle for the implementation of iNKT-based strategies against cancers, as, for instance, some reports indicate that treatments involving α-GalCer administration fail to impair tumor development [186].

Within the TME of HCC, there are various mechanisms that have been described to limit and hamper the capacity of iNKT cells to exert anti-tumor roles under normal conditions. One study reported that, in the context of HCC related to the hepatitis B virus (HBV), surrounding iNKT cells exhibited a senescence-associated phenotype, significantly impairing their ability to mount an immune response against these tumors. This imbalance in iNKT cells was attributed to an accumulation of long-chain acylcarnitines (LCAC) in the TME. Furthermore, it was seen that LCACs such as palmitoyl-carnitine and stearoyl-carnitine inhibited the expansion of iNKT cells and induced their senescence [186]. Another study in HCC reported that within the TME, there is an increase in lactic acid that reduces the expression of peroxisome proliferator-activated receptor-y (PPARy), consequently diminishing lipid biosynthesis, such as cholesterol in iNKT cells, which is required for the synthesis of IFN-γ [187]. Importantly, reduced levels of IFN-γ have been reported to be problematic for HCC patients, as this phenotype would be associated with a higher risk of tumor recurrence following treatment; hence, IFN-γ has been proposed as a relevant biomarker for a patient’s anti-tumor immunity [188]. On the other hand, it has been shown that the TME allows iNKT cells to directly exert anti-tumoral roles, particularly in ovarian cancer (OC), where iNKT were reported to be able to target the tumor through the recognition of CD1d, which was highly expressed by tumor-associated macrophages (TAMs) and myeloid-derived suppressive cells (MDSCs) [189].

Therefore, NKT cells play significant roles in defending hepatocytes and other tissues against detrimental signals that may disrupt homeostasis or promote inflammation, such as those produced during pathogen infections. Moreover, the modulation capacity of NKT cells responding to such stimuli may be negatively affected in the presence of glycolipid ligands from the tumor environment, which would impede their positive contribution in the context of anti-tumoral immune responses.

## 5. Impact of iNKT during the Establishment of Cancer and Progression

It has been described that iNKT cells can play a pivotal role in tumor immunity, either during tumor formation and/or progression [190]. Furthermore, it is noteworthy that these cells have been assessed in various types of cancer. In recent investigations using E0771 breast cancer mouse models, researchers have noted that iNKT cells, unlike the NK cells also studied, exhibit heightened activation (hyperactivation) in late-stage cancer, displaying potent effector functions within the breast tumor microenvironment. This was evidenced by the downregulation of exhaustion and inhibitory molecules (CTLA-4, TIM3, PD1) and the upregulation of activating receptors, effector molecules, and cytokines (Granzyme B, NKG2D, NKG2A, NKp46, IFN-y, IL-4, IL-17, IL-10). These findings suggest that iNKT cells could be promising candidates for tumor immunotherapy [191].

**Figure 4:**
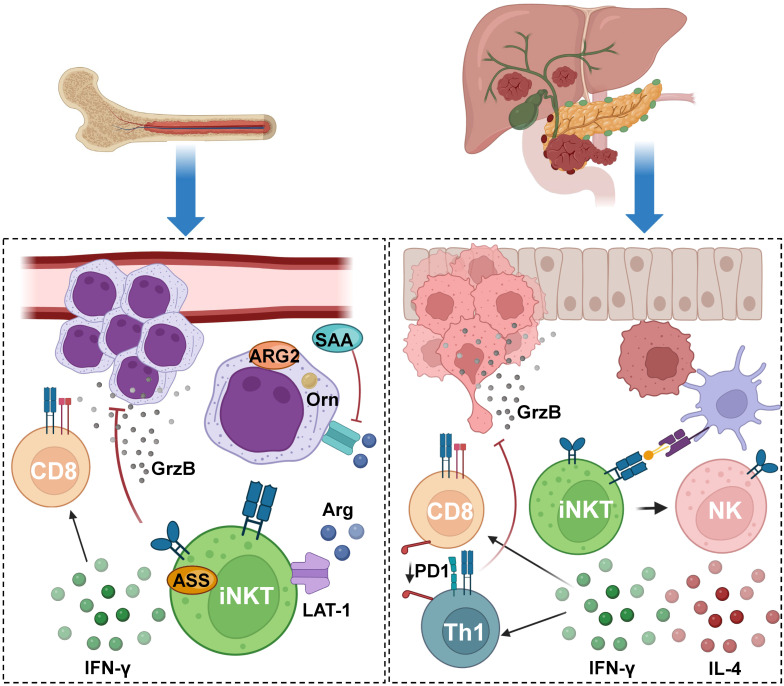
**iNKT cells response to acute myeloid leukemia and pancreatic cancer liver metastasis.** iNKT cells actively participate in tumor immunosurveillance in acute myeloid leukemia (AML), within the arginine-depleted environment induced by suppressive myeloblast through arginase 2 (ARG2), and acute-phase protein serum amyloid A (SAA). iNKT cells adapt to this environment via the upregulation of the large neutral amino acid transporter (LAT-1) and argininosuccinate synthase 1 (ASS), enabling these cells to induce a cytotoxic response. iNKT cells have also been reported to have a role in preventing metastasis progression, as described in a mouse pancreatic cancer liver metastasis (PCLM) model. iNKT cell activation with α-GalCer using APCs resulted in the suppression of PCLM progression due to increased immune cell infiltration, enhanced cytotoxic activity of iNKT and NK cells, and the promotion of a cytotoxic profile in CD4^+^ and CD8^+^ T cells. Figure created with BioRender.com.

It is known that iNKT cells actively participate in tumor immunosurveillance by restraining suppressive myeloid populations within the tumor microenvironment (Figure 4). In the case of acute myeloid leukemia (AML), these cells were documented to create an immunosuppressive milieu for conventional T cells, which usually represent a significant component of the body’s anti-tumoral response. A study found that iNKT cells adapt to the immunosuppressive environment produced by myeloblasts, which is centered around arginine depletion induced by arginase 2 (ARG2) and the release of the acute-phase protein serum amyloid A (SAA). The latter leads to increased ARG2 expression and the viability of AML myeloblasts. The adaptation of iNKT cells in this context is driven by the overactivation of the large neutral amino acid transporter (LAT-1) and argininosuccinate synthase 1 (ASS) pathways [192]. In this study, co-cultures of AML cells from diagnosed patients were conducted in the presence of α-GalCer with iNKTs alone or with T cells obtained from healthy donors. In the co-culture of iNKT cells with AML cells alone, iNKT cells induced cell death in AML cells despite the immunosuppressive AML microenvironment, primarily through iNKT cytotoxicity. In the co-culture involving T cells, iNKT cells, and AML cells, iNKT cells restored both proliferation and T cell function. Importantly, this recovery of T cell activation was found both in vitro and in a syngeneic lymphoma-bearing model. In summary, this study highlights how iNKT cells adapt to the immunosuppressive AML environment, resulting in a direct reduction in cancer burden and the restoration of T cell function, suggesting that these cells could be potential targets for treatment in this type of cancer [192].

Regarding cancer progression, another aspect to consider is metastasis, where iNKT cells also play a pivotal role. In the case of pancreatic cancer liver metastasis (PCLM), a study revealed the tumor immunity functions of iNKT cells. Researchers utilized a mouse PCLM model by injecting pancreatic tumor cells into the liver, which closely mimics clinical conditions in humans. Initially, iNKT cells were activated with α-GalCer, resulting in a significant increase in immune cell infiltration and the subsequent suppression of PCLM progression. This was demonstrated through single-cell RNA sequencing (scRNA-seq) analyses comparing immune cells from a healthy liver with a liver with PCLM in the presence or absence of α-GalCer treatment. Additionally, flow cytometry and single-cell RNA sequencing (scRNA-seq) analyses revealed enhanced cytotoxic activity of iNKT/NK cells and a shift of both CD4^+^ and CD8^+^ T cells towards a cytotoxic profile characterized by increased proliferation and downregulation of the exhaustion marker PD1. Furthermore, α-GalCer treatment exhibited reduced expression of specific markers associated with epithelial-to-mesenchymal transition and an increase in active CD4^+^ and CD8^+^ T cells. In conclusion, this work suggests that activated iNKT cells have a protective function in PCLM [193].

**Figure 5:**
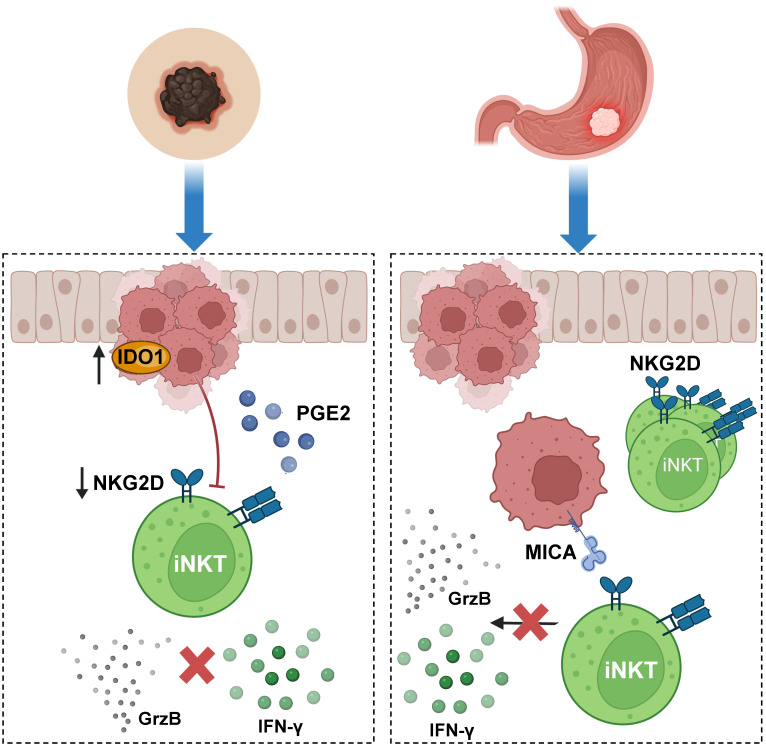
**iNKT cells display reduced function in melanoma and gastric cancer.** In a scenario related to melanoma, iNKT cell function has been reported to be suppressed in the tumor microenvironment due to cancer cells overexpressing IDO1 and secreting PGE2. Culturing iNKT cells with melanoma cell lines causes a significant reduction in the expression of the NKG2D receptor, negatively affecting the proliferation of these cells and their cytotoxic response against tumors. iNKT anti-tumoral function is also inhibited in gastric cancer (GC). In individuals with GC, the frequency and number of iNKT cells in peripheral blood are increased; however, in functional cytotoxicity assays in which iNKT cells were stimulated through the NKG2D receptor and not CD1d, these cells displayed reduced granule release and poor IFN-γ production. Figure created with BioRender.com.

Conversely, in other types of cancer, the function of iNKT cells has been found to be partially inhibited or compromised, contributing to tumor progression (Figure 5). Gastric cancer (GC) is one such example. In a study involving patients with this disease, the frequency and number of iNKT cells in peripheral blood were evaluated, along with the surface levels of NKG2D (the activating receptor) on these effector cells. The study revealed a higher frequency and number of iNKT cells in the peripheral blood of GC patients, with similar surface receptor expression levels compared to healthy donors. However, functional cytotoxicity assays involving exposure to the K562 human erythroleukemia cell line, which lacks CD1d but possesses ligands for the NKG2D receptor, showed compromised granule release and IFN-γ production by iNKT cells. This indicates that iNKT cells in GC patients have a reduced anti-tumoral response despite their increased frequency [194].

In the case of melanoma, iNKT cell function is suppressed and sometimes inhibited by melanoma cells. The investigation in this context focused on melanoma cells and their role in the tumor microenvironment (TME), which had been only partially studied. Researchers conducted co-cultures involving melanoma cell lines (A375 and WM 266-4) and iNKT cells, demonstrating a significant impact of these cell lines on the proliferation and functions of iNKT cells. This was primarily due to a significant reduction in the expression of the NKG2D receptor and the cytotoxic capacity of iNKT cells. Additionally, it was determined that melanoma cells affect the activation and functions of iNKT cells through the overexpression of IDO1 and the production of PGE2. This suppression of iNKT function was reversed when selective inhibitors targeting IDO1 and COX-2 were used [195].

In summary, iNKT cells play a dual role in tumor immunity, demonstrating an effective response in some contexts and a compromised response in others. Their adaptation to the tumor microenvironment is crucial in determining their effectiveness in cancer treatment. As such, there have been advances against metastatic cancer due to the activation of NKT through α-GalCer loaded in live attenuated bacterial vectors [196], underscoring the importance of gaining a better understanding of NKT cell anti-tumoral mechanisms to harness their therapeutic potential in cancer.

## 6. Current Advances in iNKT-Mediated Cancer Immunotherapy

Cancer immunotherapy based on iNKT cells stands out as one of the most promising cancer treatment approaches in recent times, which is supported by various studies [197,198]. Among these studies, a significant reduction in metastasis after tumor excision was found in mice transferred with dendritic cells loaded with α-GalCer. It is noteworthy that these therapies not only exert an effect through innate and adaptive immunity but also facilitate the transition from an immunosuppressive microenvironment to an immunogenic one [199,200] (Table 1).

Other investigated immunotherapies involve iNKT cells in combination with genetic engineering of hematopoietic stem cells (HSCs) collected from healthy individuals, followed by HSC-iNKT differentiation, resulting in what is referred to as AlloHSC-iNKT cells. Preclinical trials in mice have shown that these cells exhibit significant anticancer potential [201].

Various approaches have been explored in the realm of iNKT-based immunotherapies, with prominent examples being the use of ligands for these cells, such as α-GalCer, either directly associated with iNKT cells or APCs delivered through different adoptive immunotherapy methods [198,207]. For example, a phase II clinical trial showed that the administration of intravenous α-GalCer-pulsed APCs in patients with advanced or recurrent NSCLC refractory to first-line chemotherapy increased the survival of patients and was well tolerated [202]. α-GalCer has served as the cornerstone for numerous strategies aimed at developing tumor immunotherapies. Recent investigations have included the application of human iNKT cells transferred to humanized mice with tumors, along with direct α-GalCer administration into these tumors. These studies revealed a robust and rapid recruitment of iNKT cells, resulting in effective anti-tumor effects [203].

A clinical trial involving 10 patients with hepatocellular carcinoma (HCC) used adoptive transfer of iNKT cells that had been expanded and pulsed with α-GalCer. This approach led to increased Th1 cytokine production but reduced IL-4, demonstrating the safety and potential efficacy of the treatment for patients [204]. In a phase I study, APCs were pulsed with α-GalCer and subsequently administered to 21 patients via bronchoscopy. This intervention led to an increase in iNKT cells in 8 cases and IFN-γ-producing cells in 10 cases [208].

Another study evaluated the infusion of autologous iNKT during transarterial embolization (TAE) therapy in patients with HCC where transarterial chemoembolization (TACE) failed as a therapy; this approach demonstrated a significantly enhanced impact on the disease progression compared to TAE therapy alone [205].

In the context of autologous iNKT cell-based immunotherapy, a phase I clinical trial showed that adoptive transfer of iNKT that was expanded in vitro produced Th-1 responses, aiming to be an anti-tumor immunotherapy. Another phase I clinical trial revealed promising outcomes when combining iNKT cells with PD-1^+^CD8^+^ T cells in patients diagnosed with pancreatic cancer. This combined therapy showed prolonged survival for these individuals [206].

## 7. Challenges and Future Prospects of iNKT-Based Immunotherapy

Albeit the immunomodulatory effects of iNKT cells over adaptive immune cells in the tumor microenvironment following their activation have become a promising target for developing cutting-edge cancer immunotherapies, the aforementioned use of NKT cell-based strategies has faced different challenges [198], such as difficulties in acquiring strong immune responses, establishing adequate NKT phenotypes, and the delivery of NKT ligands to the TME.

Following the discovery of α-GalCer and the characterization of its anti-tumoral capabilities, initial clinical trials considered the administration of this soluble glycolipid in patients with solid tumors [209]. Although the pharmacological administration was well tolerated, improvements in the immune responses against the suppressive cells of the tumor microenvironment were not detected. Due to this observation, different hypotheses have been proposed to explain the low capacity of iNKT cells as immunotherapeutic tools in the context of cancer [210]. For instance, a significant reduction in circulating iNKT cells in prostate cancer patients, as well as lower ex vivo expansion of these cells, was found compared to cells from healthy donors [211]; furthermore, prostate cancer patient-derived iNKT cells produced lower levels of IFN-γ, proposing that the anti-tumor capacity of iNKT cells could be compromised by Th2-biased cytokine phenotypes, which was further corroborated by others [210]. On the other hand, suboptimal in vitro α-GalCer-derived activation of iNKT cells has been observed in cells obtained from patients with different types of cancers (including gastric, colorectal, gallbladder, uterus, and esophageal cancers), although these NKTs retained cytotoxic properties against tumor cells, proposing that some functional properties of iNKT cells might be impaired in cancer patients [212].

One of the biggest challenges of iNKT-based therapy against tumors comes from modulating the phenotype these cells acquire in the TME. Indeed, it has been reported that NKT1 cells displace the NKT17 phenotype and provide a response that inhibits tumor-associated M2 macrophages, reducing prostate tumor growth [213]. However, promoting the polarization toward the NKT1 phenotype in the TME has proven to be inconsistent, as evidenced by decreased IFN-γ and instead increased IL-17 secretion in mice bearing breast cancer cells [191]. Meanwhile, another issue stems from attempting to overstimulate NKT cells with α-GalCer during tumor progression, which may cause these cells to undergo anergy or promote Th2 and regulatory profiles that coincide with NKT2 and NKT10 cell subsets, which should be avoided for being detrimental to the anti-tumoral response against melanoma cells [190]. On the other hand, NKT10 cells may be beneficial when anti-inflammatory responses are required, such as preventing polyp formation in early-stage colorectal cancer [214], underscoring the importance of tailoring the phenotype of iNKTs depending on the cancer type and stage [215,216,217].

In light of the capacity of α-GalCer (KRN7000) to induce both IFN-γ and IL-4 production and the challenge presented by the opposing effects of these cytokines coupled with the unpredictable outcomes associated with α-GalCer therapies, significant efforts have been invested in synthesizing structural analogs of α-GalCer aimed at stimulating the activation and expansion of specific iNKT cell subsets [131]. Previous work has addressed the structural distinctions between α-GalCer and its derivatives, emphasizing that Th1-like analogs may address this issue [218]. This notion has been substantiated by molecules such as the AH10-7 glycolipid, as both in vivo and in vitro assays have demonstrated its ability not only to promote a polarized response dependent on IFN-γ but also to induce anti-tumoral effects in a partially humanized murine model designed to study iNKT cell responses. This model involves human CD1d expression, which replicates the frequency of iNKT cells in different tissues [84]. These findings underscore the importance of a rationalized approach in the synthesis of glycolipid ligands regarding the responses mediated by NKT cells and their implications for cancer.

Another strategy considered to enhance the therapeutic effects of iNKT cells in cancer involves the development of vehicles for the delivery of glycolipid ligands [219]. On one hand, clinical trials using α-GalCer-pulsed DCs have demonstrated a higher Th1 response compared to α-GalCer alone and strong anti-tumoral effects [220]. Further results indicate that the crucial components for the activation of naïve iNKT cells include CD1d expression and loading of NKT cell ligands, rather than the expression of co-stimulatory molecules such as CD40, CD80, and CD86 [221]. Additionally, this strategy enables the simultaneous delivery of NKT cell ligands and tumoral antigens, facilitating the activation of antigen-specific CD8^+^ T cells [222].

Recently, liposomal formulations have been broadly used for the development of vaccines and cancer treatments [223]. Interestingly, this is a somewhat cost-effective biotechnology approach consisting of unilamellar or multilamellar lipid vesicles composed of biodegradable lipids such as phosphatidylcholine and cholesterol that significantly reduce the possibility of toxic effects in the host. Furthermore, these nanoparticles are capable of encapsulating drugs in their aqueous core [223], and it is possible to anchor antigens and molecules on their surface using modified lipids, such as biotin- or nickel-functionalized liposomes. More importantly, they can capture lipophilic molecules within their lipid bilayer, such as glycolipid NKT cell ligands, promoting a strong immune response [96,224,225]. In this regard, some studies have addressed the capacity of α-GalCer-containing liposomal nanoparticles and shown that they are effective in experimental lung metastasis models, such as those using B16-F10 melanoma cells. The mechanism of action was likely related to facilitating the presentation of α-GalCer in CD1d molecules of APCs, which led to the expansion of NKT cells and decreased lung metastasis [226]. Another study showed that α-GalCer could be easily delivered to DCs and iNKT cells using liposomes containing Lewis Y, a natural glycan ligand of C-type lectin receptors, which induced a strong anti-tumoral response against the gp100/Mart-1 tumoral antigens [227]. Therefore, it would be interesting to deliver into the TME α-GalCer analogs that trigger Th1-like responses, such as AH10-7, not only to assess their capability to enhance these Th1-like responses but also to promote robust anti-tumoral activities. Importantly, such an approach has shown promising results in clinical trials [113]. Furthermore, although studies have been performed to describe the transcriptomic profiles of APCs interacting with T cells in the TME [228,229], there remains to elucidate the expression profile of NKTs in this context, thereby allowing for new strategies focused on tackling particular features of the diverse types of tumor cells to boost NKTs anti-tumoral capacity [230,231,232,233].

## 8. Concluding Remarks

Research into NKT cell subsets that elicit immunomodulatory responses in a wide array of human diseases is undoubtedly an innate immune cell type of significant interest for studying their anti-cancer potential. Furthermore, the capacity of iNKT cells to respond specifically to sphingolipid analogues such as α-GalCer and its derivatives has enabled strategies to promote their activation and differentiation toward T-helper-like phenotypes able to halt the detrimental outcomes of viral and bacterial infections for the host, which will continue to establish a niche for further studies to support their future use in clinical settings.

Moreover, since the majority of tumors employ mechanisms to evade and neutralize host anti-tumoral immunity, worsening disease prognosis and affecting the likelihood of reaching cancer metastasis, the potential that NKT cells possess, thanks to their numerous immune effectors like cytokines and receptors, may lead to cellular anti-tumoral effects. Meanwhile, on the other hand, NKT cell immune regulatory functions support the importance of these innate immune cells to boost an effective adaptive immune response against cancer formation and progression, and hence additional research may provide sustained evidence in the processes determining their involvement, to understand their critical interactions in the tumor milieu, and to provide target uses in immunotherapeutic approaches to limit or prevent cancer.

## Figures and Tables

**Table 1:** Use of iNKT-mediated cancer immunotherapy.

Cells	Cancer Cell Type (In Vitro)	Effects In Vitro	Model In Vivo	Effects In Vivo	Reference
Allogeneic Human Stem cell- Engineered iNKT (AlloHSCiNKT cells)	A375 (Melanoma), K562 (Myelogenous leukemia, H292 (Lung cancer), PC3 (Prostate cancer), MM.1S (Multiple myeloma)	Reduction in metastasis, enhanced tumor killing efficacy	Human melanoma xenografth NSG mouse	Suppresed tumor growth	Ruide Li et al., 2021 [201]
α-GalCer-pulsed APCs to activate iNKT cells	-	-	NSCLC	Decreased iNKT and increase in NK cells, interferon-γ-producing cells and effector CD8^+^ T cells	Toyoda et al., 2020 [202]
PBMC-iNKT	A375-CD1d-FG and H292-CD1d-FG	Enhanced tumor cell killing and cytotoxic function	Human lung cancer xenograft NSG mouse	Enhanced antitumor ability	Ruide Li et al., 2022 [203]
			Hepatocellular carcinoma	Greater production of Th1 cytokines and less IL-4. Safe and well tolerated treatment	Gao et al., 2021 [204]
			Unresectable hepatocellular carcinoma after TACE failure	Showed improvements in the development of the disease in comparation to TAE treatment alone	Guo et al., 2023 [205]
			Stage IV Pancreatic Cancer	Safe treatment, prolonged survival	Wang et al., 2023 [206]

## Data Availability

No new data were created or analyzed in this study. Data sharing is not applicable to this article.
